# Paranormal belief, cognitive-perceptual factors, and well-being: A network analysis

**DOI:** 10.3389/fpsyg.2022.967823

**Published:** 2022-09-15

**Authors:** Neil Dagnall, Andrew Denovan, Kenneth G. Drinkwater

**Affiliations:** ^1^Department of Psychology, Manchester Metropolitan University, Manchester, United Kingdom; ^2^Department of Psychology, University of Huddersfield, Huddersfield, United Kingdom

**Keywords:** manic-depressive experience, network analysis, paranormal belief, schizotypy, transliminality, well-being

## Abstract

By assessing interrelationships among variables within a specified theoretical framework, network analysis (NA) provides nuanced insights into how associations between psychological constructs are related to outcome measures. Noting this, the authors used NA to examine connections between Paranormal Belief, cognitive-perceptual factors (Schizotypy, Transliminality, and Manic-Depressive Experience), and well-being (Life Satisfaction, Meaning in Life, Somatic Complaints, Perceived Stress, Depressive Symptoms). Data derived from a sample of 3,090 participants (mean age = 50.30, standard deviation = 15.20; 46.5% male, 53.1% female) who completed standardised self-report measures capturing the study constructs online. Transliminality, Unusual Experiences (positive schizotypy), and Depressive Experience demonstrated high expected influence centrality. This indicated that these factors were the most strongly connected and influential in the network. Moreover, Transliminality was a connecting variable between Paranormal Belief, positive schizotypy, and psychopathology. Depressive Experience bridged the relationship between Transliminality and well-being. The conceptual implications of these outcomes are discussed with regards to better understanding relationships between Paranormal Belief, cognitive-perceptual factors, and well-being.

## Introduction

Historically, investigators have reported positive correlations between paranormal belief and psychopathological indicators. These include, but are not restricted to, higher scores on manic (Thalbourne and French, 1995), depressive (Thalbourne and French, 1995), and psychotic (Dag, 1999) scales. Such findings imply that supernatural credence is a prognosticator of poorer psychological adjustment and reduced well-being (e.g., heightened negative emotional states and depressive attributional style) (Dudley and Whisnand, 2000). Indeed, Pérez Navarro and Martínez Guerra (2020) recently established that psychopathology-related constructs (i.e., schizotypy) were significantly associated with paranormal belief. Caution is required here, however, because schizotypy was assessed as a global construct, whereas current schizotypy models depict this as multidimensional (Grant et al., 2018).

The negative relationship between paranormal belief and well-being is also demonstrated via superstition, a facet of belief that refers to the conviction that real-world events are affected by supernatural agencies such as luck, destiny, and fate. Illustratively, superstition is associated with lower self-efficacy (Tobacyk and Shrader, 1991), higher trait anxiety (Wolfradt, 1997) (see Dagnall et al., 2007, 2009), and external locus of control (Hoffmann et al., 2022). Based on such findings several theorists conclude that paranormal belief is a manifestation of/or concomitant with non-adaptive psychological processes (Irwin, 1993, 2000, 2009).

Consistent with this interpretation, Irwin (1993) proposed the psychodynamic functions hypothesis, which views paranormal beliefs as needs-serving attempts to resolve ambiguity. Explicitly, belief bestows meaning upon the world, and in doing so affords a personal sense of reassurance. For instance, Keinan (1994) reported during the Gulf War residents living in high-stress locations (vs. low stress) scored higher on magical thinking. A further illustration is Padgett and Jorgenson (1982), who observed that measures of economic threat were directly related to elevated levels of superstition in Germany between 1918 and 1940.

Although, paranormal beliefs are beneficial to the extent that they provide an illusion of control within particular situations, Irwin contends they are actually characteristic of ineffective coping (Irwin, 1993, 2000, 2009). This is because psychological benefits are domain specific; fail to extrapolate across situations (Roe and Bell, 2016). Thus, paranormal belief generally signifies an inability to adequately address demanding circumstances. In this context, belief promotes avoidance and prevents engagement with goal-related behaviours (i.e., approach coping) (Marchlewska et al., 2022). Approach coping is important to well-being as the adoption of active strategies (e.g., solution focussed orientation, acceptance of challenge, and engagement with social support) facilitates positive affect (Fortune et al., 2002). Aligning with this notion, functional disability and depression correlate negatively with approach coping (Greenglass et al., 2006). Thus, from the psychodynamic perspective, paranormal belief represents inadequate stress management (Mackay et al., 2011). This supposition concurs with the negative correlation that Irwin (1991) observed between paranormal belief and psychological coping. Moreover, McGarry and Newberry (1981) found that believers typically regarded the world as problematic, unfair, and unpredictable, as echoed by Stone (2016).

Though some evidence suggests paranormal belief is associated with higher levels of psychopathology and lower well-being, findings overall are inconsistent. For example, Yamane et al. (2019) found that paranormal belief was positively associated with increased happiness (well-being), and Haider (2019) found that paranormal belief influenced life satisfaction (well-being) via happiness orientation. In addition, Schumaker (1987) reported a negative correlation with severity of psychopathology. Noting this, Schumaker (1990) suggested that belief performs an adaptive function by sheltering individuals from reality. This, however, has proved difficult to replicate, and Irwin (1991) produced contradictory results. Additionally, several researchers have failed to observe significant relationships between paranormal belief and well-being (Willging and Lester, 1997). These varying outcomes, in part, may be attributable to the use of different paranormal and well-being measures.

Furthermore, the contention that paranormal belief is a direct indicator of poor psychological functioning conflicts with the high prevalence of supernatural endorsement (i.e., belief, experience, ability, and engagement) in non-clinical populations (Dagnall et al., 2016). Indeed, recent work advises that paranormal belief is only allied to poor psychological functioning in certain circumstances. For example, Dagnall et al. (2022a) found that higher belief was only associated with reduced well-being (i.e., higher perceived stress and somatic complaints, and lower life satisfaction) when it co-occurred with higher levels of psychopathology.

This suggests that inter-relationships between belief and cognitive-perceptual factors determine outcome. Indeed, Dagnall et al. (2022b) reported that paranormal belief was only associated with lower well-being when belief interacted with schizotypy subfactors (Unusual Experiences and Cognitive Disorganisation), transliminality, and manic-depressive experience. The importance of these affiliated variables is demonstrated via consideration of their nature. Unusual Experiences comprises positive, productive features of psychosis (i.e., perceptual aberrations, magical thinking, and hallucinations), and Cognitive Disorganisation denotes disorganised elements (e.g., poor attention/concentration) (Mason et al., 2005). Transliminality signifies hypersensitivity to psychological material (originating in the unconscious, and/or the external environment) (Thalbourne, 1998), and manic-depressive experiences reflect the degree to which individuals undergo extremely heightened and decreased arousal, affect, and energy (Thalbourne and French, 1995).

Paranormal belief in the absence of these cognitive-perceptual factors is relatively benign and largely unrelated to well-being (Williams et al., 2021). Certainly, paranormal beliefs are typically only problematic when they disrupt everyday functioning. This is generally not the case since surveys robustly report that high percentages of participants within general samples believe in and report experience of paranormal phenomena (Dagnall et al., 2015).

The hypothesis that paranormal belief has an indirect effect on negative well-being, via positive associations with cognitive-perceptual factors, which are more strongly predictive of mental functioning, concurs with recent academic work and explains why many previous studies report weak/moderate correlations between paranormal belief and negative well-being (Dagnall et al., 2022a,b).

### Network analysis

Acknowledging these points, the present paper used network analysis (NA) to examine relationships between paranormal belief, cognitive-perceptual factors, and well-being. NA is a methodology derived from network science that enables simultaneous consideration of multiple interacting factors within a mathematical model (Levinson et al., 2017). This approach places an emphasis on the recognition of system components (nodes) and the strength of associations between them (links). NA represents phenomena within a network, where variables appear as nodes and pairwise conditional associations between factors are signified by edges (Borsboom et al., 2021). This visual representation encapsulates both relationships among variables and underlying structural similarity. NA is important because it recognises that consideration of components in isolation provides only limited theoretical insights.

A benefit of NA over traditional statistical procedures is that it allows researchers to recognise patterns within data (i.e., visualisation of weighted edges as correlation strength permits identification of structures). The ability to conceptualise complex statistical patterns without employing data reduction techniques is a powerful analytical tool (Heshmati et al., 2020). Moreover, NA is a significant approach because it enables investigators to identify important nodes. These are features, which based on connections, greatly influence the network. Such nodes specify the extent to which the network remains connected if the variable is removed (system tolerance). This information is important theoretically (i.e., informs model development) and practically (i.e., indicates which variables may provide useful interventions) (Valente, 2012; Heshmati et al., 2020).

The application of network approaches to academic fields has extended scholarly understanding and analytical sophistication (Borsboom et al., 2021). For instance, in health-related disciplines, the use of NA has allowed researchers to investigate how complex interactions between psychological and biological factors influence perceptions of health and well-being. This is especially true in psychopathology, where NA has become increasingly popular (Jones et al., 2018). NA is particularly attractive in this domain as it produces models that possess the potential to improve comprehension and treatment of disorders. This is important because investigators have traditionally assumed that symptoms arise from a common cause (i.e., a latent variable). In contrast to monocausal interpretation, the underlying assumption of NA is that symptoms influence and interact (Borsboom and Cramer, 2013).

Furthermore, NA (using measures of centrality) can categorise fundamental features, which define phenomena under observation. In this context, within networks psychopathological symptoms represent integral components of the conditional system, which produce, maintain, and underlie disorder (Levinson et al., 2017). Accordingly, interrelations strengthen or weaken psychopathological adjustment. In the case of strongly correlated nodes these form interlinked communities, where each node has unique relationships with nodes in other communities. The ability to extend beyond association to identification of interacting and/or reciprocally reinforcing factors explains why NA is regarded as a powerful analytical technique.

### The present study

Despite the potential of NA, researchers have yet to apply it to relationships between paranormal belief, cognitive-perceptual factors, and well-being. Its implementation is important because it allows researchers to determine the extent to which beliefs are central to well-being, and it reveals the importance of interactions with cognitive-perceptual factors. Thus, NA provides a nuanced understanding of how paranormal belief and cognitive-perceptual factors relate to one another and how they might impact well-being. This can potentially identify the combination(s) of paranormal belief and cognitive-perceptual factors that most affect well-being and inform the development of interventions. Therefore, the specific objectives of this study were: (1) to examine connections between belief in the paranormal, cognitive-perceptual factors, and well-being indices using NA; and (2) to explore which variables are central to this relationship/network.

Based on past literature (e.g., Dagnall et al., 2016; Williams et al., 2021), the authors hypothesised that paranormal belief would play a minor role in the network, whereas cognitive-perceptual factors would be central to well-being facets (and vice versa). Finally, paranormal belief was predicted to be most strongly related to transliminality and schizotypy (Unusual Experiences), supporting its role in providing a framework for structuring odd and unusual mentation.

## Materials and methods

### Participants

A total of 3,090 participants (*M*age = 50.30, *SD* = 15.20, range 18–91) participated in this study. The sample comprised 1,436 males (46.5%; *M*age = 54.95, *SD* = 14.59, range 18–88), 1,642 females (53.1%; *M*age = 46.27, *SD* = 14.55, range 18–91), and 12 non-binary participants (0.4%; *M*age = 44.50, *SD* = 16.61, range 25–71). Participants were recruited via Bilendi, a supplier of representative online samples (Salak et al., 2021). Research indicates that panel data is equivalent to traditional collection approaches (Kees et al., 2017). The authors specified a representative United Kingdom-based sample, with equal gender, and a minimum age of 18 years.

### Measures

#### The Revised Paranormal Belief Scale

The Revised Paranormal Belief Scale (RPBS) (Tobacyk, 2004) is an established measure of belief in the paranormal. It includes 26-items (e.g., “There is a devil”) completed using a 7-point response format (1 = strongly disagree to 7 = strongly agree). Consistent with Irwin (2009) scores were converted to 0 to 6. The RPBS possesses satisfactory validity and reliability (Drinkwater et al., 2017). Excellent reliability was found in this study, α = 0.96.

### Cognitive-perceptual measures

#### The Oxford-Liverpool Inventory of Feelings and Experiences

The Oxford-Liverpool Inventory of Feelings and Experiences (O-LIFEshort) (Mason et al., 2005) is a 43-item version of the O-LIFE (Mason et al., 1995). The scale assesses schizotypal characteristics among non-clinical samples. It comprises four subscales: Unusual Experiences, Cognitive Disorganisation, Introvertive Anhedonia, and Impulsive Non-Conformity. Unusual Experiences (12-items) assesses positive schizotypy (magical thinking, perceptual anomalies). Cognitive Disorganisation (11-items) measures disorganised aspects of psychosis such as deficient concentration/attention. Introvertive Anhedonia (10-items) indexes negative schizotypy (e.g., intimacy avoidance, withdrawal). Impulsive Non-Conformity (10-items) captures antisocial tendencies and deficiencies in self-control. Items (e.g., “Are you a person whose mood goes up and down easily?”) include a “yes/no” format. Subscale alpha reliability ranges from 0.62 to 0.80 (Mason et al., 2005). In this study, reliability (Unusual Experiences α = 0.86, Cognitive Disorganisation α = 0.86, Introvertive Anhedonia α = 0.61, Impulsive Non-Conformity α = 0.66) was comparable with previous research (e.g., Mason et al., 2005).

#### Manic-Depressiveness Scale

The Manic-Depressiveness Scale (Thalbourne et al., 1994) includes two 9-item “true/false” subscales: Manic Experience (e.g., “I have sometimes behaved in a much more impulsive or uninhibited way than is usual for me”), and Depressive Experience (e.g., “I have on at least one occasion felt that there was no purpose in life”). The measure has established validity and reliability (see Lester, 2000). Satisfactory reliability was observed within this investigation (Manic Experience α = 0.74, Depressive Experience α = 0.80).

#### The Revised Transliminality Scale

The Revised Transliminality Scale (RTS) is a Rasch scaled version of Thalbourne (1998) original 29-item scale (Lange et al., 2000). Items (e.g., “I sometimes have a feeling of gaining or losing energy when certain people look at me or touch me”) are accompanied by a “yes/no” format. Although all items are administered, 12 are excluded due to age and gender bias (see Houran et al., 2003). The measure possesses adequate to good reliability (Houran et al., 2003), which was also evident in this study, α = 0.88.

### Well-being

#### The 10-item Perceived Stress Scale

The Perceived Stress Scale (PSS-10) (Cohen and Williamson, 1988) assesses individual perceptions of stress in the past month. The measure presents items as statements (e.g., “How often have you felt nervous and stressed?”), and participants answer using a 0 (never) to 4 (very often) format. Previous research indicates that the PSS-10 has satisfactory reliability and validity (Denovan et al., 2019). Good reliability was evident in this study, α = 0.87.

#### The Somatic Symptom Scale-8

The Somatic Symptom Scale-8 (SSS-8) (Gierk et al., 2014) examines susceptibility to somatic complaints (e.g., “Dizziness”). Items index the extent to which somatic burdens have affected participants using a seven-day timeframe. A 5-point scale accompanies items, from 0 (not at all) to 4 (very much). The measure has good internal reliability (Gierk et al., 2014), which was observed in this study, α = 0.87.

#### Center for Epidemiologic Studies-Depression Scale

The Center for Epidemiologic Studies-Depression Scale (CES-D) (Radloff, 1977) is an established measure of depressive symptoms. The scale comprises 20-items and a response format of 0 (rarely) to 3 (most or all of the time). Items (e.g., “I felt sad”) concentrate on the previous week. The CES-D has demonstrated high internal reliability (Hann et al., 1999). Within this study, good reliability was found, α = 0.85.

#### The Satisfaction with Life Scale

The Satisfaction with Life Scale (SWLS) (Diener et al., 1985) assesses subjective well-being. The instrument comprises 5-items (e.g., “I am satisfied with my life”), which are presented alongside a seven-point response scale from 1 (strongly disagree) to 7 (strongly agree). The SWLS has consistently displayed high internal consistency (Diener et al., 1985). This occurred also in this study, α = 0.92.

#### The Meaning in Life Questionnaire

The Meaning in Life Questionnaire (MLQ) (Steger et al., 2006) captures meaning in life as an aspect of well-being by focussing on the degree to which participants feel their life is significant and has a purpose. The scale consists of 10-items (e.g., “My life has a clear sense of purpose”) with a 7-point response scale, ranging from 1 (Absolutely Untrue) to 7 (Absolutely True). Across the literature the scale demonstrates excellent reliability (Steger et al., 2006). Within this paper, the researchers observed good reliability, α = 0.83.

### Procedure

Participants accessed study information by clicking on a web link. Only individuals who met the inclusion criteria and provided informed consent progressed to the survey. This comprised a demographics section (age and preferred gender) and the self-report measures. Rotation of sections across participants controlled for order effects. To address social desirability participants were told that there were no correct answers. To limit common method variance instructions encouraged psychological separation by stressing differences between constructs (Krishnaveni and Deepa, 2013). Participants were debriefed after completing the measures.

### Ethics statement

The Manchester Metropolitan University Faculty of Health, Psychology and Social Care Ethics Committee granted ethical approval (December 2020; Project ID, 25390).

### Analysis

Following data screening, assessment of descriptive statistics and Bayesian Pearson correlations a network comprising Paranormal Belief, cognitive-perceptual characteristics, and well-being was estimated. A graphical least absolute shrinkage and selection operator (Friedman et al., 2008) was performed using JASP. This was based on the Extended Bayesian Information Criterion (EBIC; Chen and Chen, 2008) called the EBICglasso model.

To achieve a parsimonious network, the tuning parameter was set at 0.5. The centrality of variables was assessed using betweenness, closeness, strength (degree), and expected influence. The authors focussed on strength because it reflected the most important nodes (variables) alongside reasonably accurate centrality estimates (Santos et al., 2018). In addition, standardised expected influence was considered as this infers the sum of all node edges.

To test stability of central indices, case-dropping bootstrapping (Epskamp and Fried, 2018) was utilised. This computes a correlation between the original centrality indices and the correlation from a data subset. Stability should be greater than 0.7. The accuracy of edge weights was determined via bootstrap 95% confidence intervals (CIs) (Epskamp et al., 2018). Narrower CIs are desirable. For both bootstrap analyses, 1,000 resamples were specified.

## Results

### Descriptive statistics and correlation analyses

Data screening (*N* = 3103) revealed 13 data points <-3.25 or >3.25. Consistent with Tabachnick et al. (2007) these were removed. Skewness and kurtosis fell between –2 and +2 and are presented alongside means and standard deviations (Supplementary Appendix 1). As shown in Table 1, significant positive correlations existed among Paranormal Belief, cognitive-perceptual factors (O-LIFEshort subscales, Manic-Depressive Experience, and Transliminality), Depressive Symptoms, Perceived Stress, and Somatic Complaints. Life Satisfaction exhibited significant negative associations with all variables aside from Paranormal Belief (non-significant) and Meaning in Life (significant positive correlation). Meaning in Life displayed mixed relationships with the other study variables. Specifically, positive and significant (<0.001) with Paranormal Belief, Unusual Experiences, Transliminality, and Life Satisfaction; negative and significant (<0.001) with Introvertive Anhedonia; and weakly associated (>0.001) with the remaining measures. The Bayesian correlation test revealed that most of log(BF_10_) were greater than 3, supporting the significance/strength of relationships.

**TABLE 1:** Correlations among study variables.

Variable	1	2	3	4	5	6	7	8	9	10	11	12	13
1. Paranormal Belief		0.54**	0.31**	0.05	0.31**	0.30**	0.27**	0.30**	0.50**	0.26**	0.33**	–0.03	0.32**
log(BF_10_)		538.05	147.13	0.49	151.10	140.05	110.79	142.11	434.55	106.29	169.27	–2.52	157.98
2. Unusual Experiences			0.60**	0.07**	0.49**	0.45**	0.55**	0.54**	0.73**	0.36**	0.41**	–0.10**	0.21**
log(BF_10_)			∞	3.93	422.10	353.18	549.69	520.06	∞	204.28	283.17	10.84	63.22
3. Cognitive Disorganisation				0.23**	0.51**	0.51**	0.54**	0.59**	0.52**	0.53**	0.44**	–0.29**	0.00
log(BF_10_)				79.31	458.88	468.89	537.85	∞	478.06	501.64	326.48	128.76	–3.78
4. Introvertive Anhedonia					0.21**	0.17**	0.09**	0.21**	0.02	0.28**	0.22**	–0.30**	–0.25**
log(BF_10_)					68.32	42.64	9.79	65.86	–3.06	122.66	70.11	138.27	96.12
5. Impulsive Non-Conformity						0.48**	0.49**	0.58**	0.47**	0.48**	0.43**	–0.26**	0.01
log(BF_10_)						407.70	411.74	∞	386.46	407.63	314.86	107.23	–3.76
6. Depressive symptoms							0.47**	0.55**	0.43**	0.55**	0.59**	–0.28**	0.05*
log(BF_10_)							388.91	557.18	314.17	545.37	∞	118.09	–0.73
7. Manic Experience								0.69**	0.64**	0.37**	0.44**	–0.17**	0.04*
log(BF_10_)								∞	∞	222.47	321.18	42.53	–1.87
8. Depressive Experience									0.59**	0.55**	0.53**	–0.35**	–0.01
log(BF_10_)									∞	539.26	510.96	202.26	–3.75
9. Transliminality										0.32**	0.41**	–0.07**	0.23**
log(BF_10_)										158.21	283.59	4.05	79.18
10. Perceived Stress											0.53**	–0.55**	–0.04*
log(BF_10_)											504.39	539.39	–1.38
11. Somatic Complaints												–0.29**	0.01
log(BF_10_)												127.43	–3.75
12. Life Satisfaction													0.37**
log(BF_10_)													217.10
13. Meaning in Life													
log(BF_10_)													

*p < 0.05; **p < 0.001.

### EBICglasso network analysis

**FIGURE 1:**
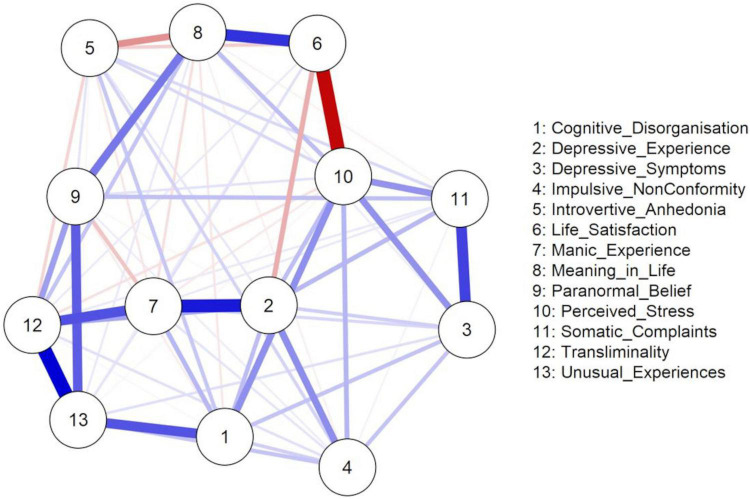
EBICglasso model based on the network analysis of the relationships between Paranormal Belief, cognitive-perceptual factors, and well-being variables.

The EBICglasso network depicting relations among Paranormal Belief, cognitive-perceptual factors, and well-being appears in Figure 1. Blue and red edges represent positive and negative associations respectively, and thicker edges portray stronger relationships. There were 13 nodes and 69 non-zero edges. The network was interconnected, indicating strong edges among Paranormal Belief and cognitive-perceptual factors (particularly Unusual Experiences, Cognitive Disorganisation, Transliminality, Manic-Depressive Experience). Moreover, strong edges existed among well-being factors (Depressive Symptoms, Perceived Stress, Life Satisfaction, Somatic Complaints, Meaning in Life).

The weights matrix (Supplementary Appendix 2) supported these results. Relationships among well-being, Paranormal Belief, and cognitive-perceptual factors, reinforced the observation of a positive link between Meaning in Life, Paranormal Belief and Transliminality, and a negative link between Meaning in Life and Introvertive Anhedonia. A positive relationship existed between Perceived Stress and Impulsive Non-Conformity, Cognitive Disorganisation, and Depressive Experience. Life Satisfaction evidenced a notable negative relationship with Depressive Experience, whereas Somatic Complaints displayed positive relations with Paranormal Belief and Depressive Experience. Remaining relationships between well-being variables and Paranormal Belief and cognitive-perceptual factors were weaker. The accuracy of the edge weights, estimated using bootstrap 95% non-parametric CIs, is shown in Supplementary Appendix 3. The 95% CIs were narrow, and the majority did not cross zero, indicating that edges were trustworthy.

**FIGURE 2:**
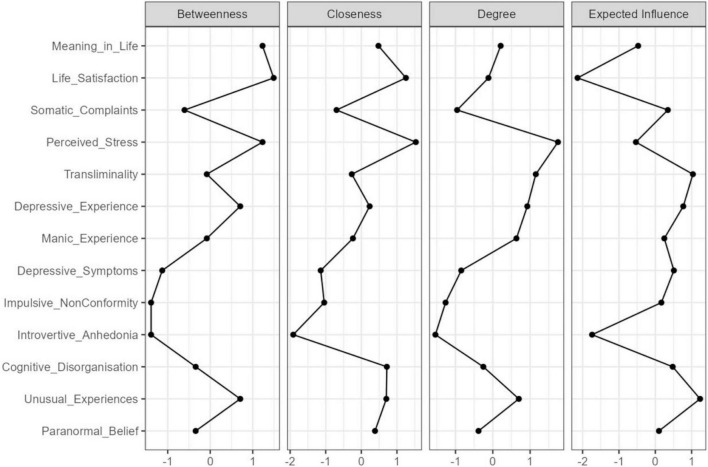
Centrality plots for EBICglasso network depicting the betweenness, closeness, degree (strength), and expected influence of each node (variable).

The standardised estimates of the centrality indices for betweenness, closeness, strength (degree), and expected influence are presented in Table 2. To ease interpretation, plots for the centrality measures in terms of z-scores are displayed in Figure 2. Centrality indices (i.e., strength and expected influence) were considered. Nodes with the highest strength centrality values were Transliminality, Perceived Stress, Depressive Experience, and Unusual Experiences. Nodes with the lowest strength were Impulsive Non-Conformity, Introvertive Anhedonia, and Somatic Complaints. Unusual Experiences, Transliminality, and Depressive Experience possessed the greatest standardised expected influence. Life Satisfaction and Introvertive Anhedonia possessed the lowest standardised expected influence. Based on associations and the centrality of Transliminality, it appears that the construct bridges/connects Paranormal Belief and Unusual Experiences with psychopathology variables such as Manic-Depressive Experience. Also, Depressive Experience may bridge/connect Transliminality and schizotypy with well-being variables (i.e., Perceived Stress, Somatic Complaints and Life Satisfaction) (Figure 1).

**TABLE 2:** Centrality measures.

Variable	Betweenness	Closeness	Strength (Degree)	Expected influence
Cognitive Disorganisation	–0.34	0.72	–0.26	0.48
Depressive Experience	0.71	0.23	0.93	0.77
Depressive Symptoms	–1.13	–1.14	–0.85	0.51
Impulsive Non-Conformity	–1.39	–1.04	–1.27	0.17
Introvertive Anhedonia	–1.39	–1.91	–1.54	–1.74
Life Satisfaction	1.49	1.25	–0.12	–2.14
Manic Experience	–0.10	–0.24	0.63	0.25
Meaning in Life	1.23	0.48	0.21	–0.47
Paranormal Belief	–0.34	0.38	–0.38	0.10
Perceived Stress	1.23	1.53	1.75	–0.53
Somatic Complaints	–0.61	–0.69	–0.96	0.35
Transliminality	–0.08	–0.27	1.15	1.03
Unusual Experiences	0.71	0.70	0.69	1.23

The stability of the central indices examined using case-dropping bootstrapping is shown in Supplementary Appendix 4. For all indices, notably strength, the correlation stability coefficient remained above 0.7, indicating satisfactory stability (Epskamp et al., 2018).

## Discussion

Network analysis (NA) revealed that the central model variables were Transliminality, Perceived Stress, Depressive Experience, and Unusual Experiences. Paranormal Belief was not primarily involved in the psychopathology-well-being relationship. These findings aligned with recent research, reporting that belief in the absence of high scores on cognitive-perceptual factors (i.e., schizotypy) is benign (Dagnall et al., 2022a,b). As predicted Paranormal Belief was most strongly associated with Unusual Experiences (positive schizotypy) and Transliminality. Positive schizotypy is important because it extends “normal” experience by facilitating the production of “additional” thoughts and feelings (Fleck et al., 2008; Barrantes-Vidal et al., 2013), and high transliminality is integral because it indicates acute stimuli sensitivity (Dagnall et al., 2008, 2010). Accordingly, results supported the proposition that paranormal belief provides a framework for interpretating odd/unusual mentation. The positive relationship between Paranormal Belief and Unusual Experiences further aligned with this notion. By controlling for the influence of other variables, NA reinforced this conclusion.

Within paranormal believers, Transliminality and psychopathology-related variables increase propensity to experience negative well-being outcomes (Dagnall et al., 2022b). In this study, Transliminality performed as a “bridging node,” potentially acting as a causal pathway connecting Paranormal Belief and positive schizotypy with psychopathological variables. This interpretation is commensurate with the conceptualisation of transliminality as involuntary susceptibility to ideational and affective phenomena that increases the likelihood of experiencing clinical depression, mania, and magical ideation. Correspondingly, individuals higher in transliminality believe more in the paranormal (Thalbourne and Houran, 2000). The notion that this connects Paranormal Belief and positive schizotypy with manic-depressive experience aligns with the supposition that Transliminality is a trait denoting vulnerability to psychopathology. This relationship is explained by characteristics such as hypersensitivity, reduced latent inhibition (Carson, 2011), and lower cognitive flexibility (Peters et al., 1999), which are features of psychosis (see Williams et al., 1998; Waltz, 2017; Calvo et al., 2021).

Depressive Experience acted as a bridge node connecting Transliminality with negative well-being features (lower Life Satisfaction, higher Perceived Stress, and Somatic Complaints), and partially connected schizotypy (particularly Cognitive Disorganisation and Impulsive Non-Conformity, which occupied direct links as well). Cognitive Disorganisation is associated with distressing experiences (e.g., Schofield and Claridge, 2007), whereas individuals reporting higher Impulsive Non-Conformity typically report depressive symptoms (Claridge and Blakey, 2009). It is possible that Depressive Experience connected schizotypy and Transliminality with negative well-being via negative affect. Explicitly, Abbott et al. (2012) postulate that negative affect, a central component of depression (Cohen et al., 2017), is a key factor associating diminished well-being with schizotypy.

It is likely that Paranormal Belief relates negatively to well-being indirectly via Transliminality. Future research should examine this because the cross-sectional design prohibits causal understanding. Paranormal Belief evidenced a stronger (direct) relationship with Meaning in Life. This is supported by previous work (Irwin, 1993; FioRito et al., 2021), which indicates that believers are more likely to be motivated by the need for explanation.

### Limitations of network analysis

Data and conclusions were limited by the variables included within the network. Other factors may produce different psychopathology models. Noting this, subsequent research should replicate and extend the current study using associated measures. This is especially important given several of the relationships demonstrated weak to moderate interrelationships. Additionally, ensuing work should test the stability of outcomes across different samples. This is vital since it will establish the robustness of centrality indices.

Despite increased interest in and the application of NA, the approach applied to psychopathology is relatively new. Hence, criteria such as establishment of fit indices and tests of reliability are still evolving. Thus, though NA provides important clinical insights it is necessary to validate these by comparing findings with traditional methods such as multidimensional models of psychopathology and latent variable analysis (e.g., Prisciandaro and Roberts, 2009). Notwithstanding the confines of NA, this paper demonstrated how its application to paranormal belief can enhance theoretical understanding and inform the development of nuanced models of psychological adjustment and well-being. Explicitly, NA suggests ways in which cognitive-perceptual factors interact to affect positive and negative outcomes.

A more general limitation was that the study was cross-sectional. Hence, although NA identified potential dynamic relationships between factors causation cannot be inferred. To achieve this, longitudinal and/or experimental data are required. The use of controlled multiple time points will help to determine the extent variables are causally related and whether these relationships change over time. In addition, NA did not establish direction among the variables, and it is possible that well-being, Paranormal Belief, and cognitive-perceptual factors occupy a self-reinforcing relationship. However, direction was indicated throughout this research (in terms of Paranormal Belief and cognitive-perceptual factors → well-being) because constructs (i.e., schizotypy and Transliminality) are trait-based (see Ericson et al., 2011), whereas well-being variables are often considered as consequent (e.g., Dagnall et al., 2022b).

Finally, findings require cautious interpretation because they are exploratory and paranormal beliefs generally are not clinical in nature. Like schizotypy they are best conceptualised on the normal-to-pathological continuum. Hence, many paranormal beliefs and experiences are benign (Grant and Hennig, 2020). From this perspective, like healthy schizotypes who experience positive schizotypy and affective features that map onto bipolar disorder (see Mohr and Claridge, 2015), some believers/experiencers are well individuals who exhibit psychotic-like traits.

These believers use supernatural credence as an adaptive cognitive framework for making sense of the world. Explicitly, belief helps then to structure, integrate, and comprehend their unusual perceptions, cognitions, and experiences. Accordingly, subsequent research should investigate which beliefs are adaptive, benign, and harmful to well-being and identify associated factors. Key to this is not only the content of the belief but also consideration of the impact it has on the individual (O’Keeffe et al., 2019). This is important since similar beliefs can affect believers in different ways depending upon their attributions. Investigating these factors will further extend understanding of the nature of paranormal belief.

### Conclusion

Overall, results indicated that Transliminality, positive schizotypy, and Depressive Experience were core nodes in the network. Transliminality bridged the Paranormal Belief, positive schizotypy, and psychopathology relationship, whereas Depressive Experience bridged the relationship between Transliminality and well-being. Transliminality and Depressive Experience potentially provided a causal pathway, elucidating how Paranormal Belief relates to well-being. It is important for future research to test this finding using longitudinal and/or experimental data.

## Data availability statement

The raw data supporting the conclusions of this article will be made available by the authors, without undue reservation.

## Ethics statement

The studies involving human participants were reviewed and approved by the Manchester Metropolitan University Faculty of Health, Psychology and Social Care Ethics Committee (December 2020; Project ID, 25390). Written informed consent for participation was not required for this study in accordance with the national legislation and the institutional requirements. Written informed consent was obtained from the individual(s) for the publication of any potentially identifiable images or data included in this article.

## Author contributions

AD and ND designed the study. ND provided conceptual input and developed the theoretical context for the study, summarised findings and edited all sections. AD collated measurement scales, arranged data collection, and undertook analyses. KD and AD revised the final manuscript and prepared the draft submission. All authors contributed meaningfully to the article and approved the final version.
