# Supplementary material for: Paranormal belief, cognitive-perceptual factors, and well-being: A network analysis
Source: Front Psychol. 2022 Sep 15;13:967823. doi: 10.3389/fpsyg.2022.967823 (PMC9521162; doi:10.3389/fpsyg.2022.967823)
Supplement: Supplementary file 1 [file Table_1.docx]

Appendix S1. Descriptive information for all study variables

| Variable | Mean | *SD* | Skewness | Kurtosis |
| --- | --- | --- | --- | --- |
| Paranormal Belief | 57.68 | 33.71 | .06 | -.81 |
| Unusual Experiences | 3.41 | 3.29 | .77 | -.37 |
| Cognitive Disorganisation | 3.96 | 3.41 | .55 | -.83 |
| Introvertive Anhedonia | 3.47 | 2.12 | .16 | -.71 |
| Impulsive Non-Conformity | 2.51 | 2.12 | .77 | -.14 |
| Depressive symptoms | 18.23 | 9.62 | .78 | .33 |
| Manic Experience | 3.00 | 2.36 | .50 | -.61 |
| Depressive Experience | 2.70 | 2.46 | .69 | -.49 |
| Transliminality | 20.06 | 5.28 | .71 | .39 |
| Perceived Stress | 16.65 | 7.85 | .12 | -.20 |
| Somatic Complaints | 16.63 | 7.14 | .79 | -.07 |
| Life Satisfaction | 20.61 | 7.38 | -.35 | -.59 |
| Meaning in Life | 39.65 | 10.26 | -.43 | .67 |
