# Supplementary material for: Paranormal belief, cognitive-perceptual factors, and well-being: A network analysis
Source: Front Psychol. 2022 Sep 15;13:967823. doi: 10.3389/fpsyg.2022.967823 (PMC9521162; doi:10.3389/fpsyg.2022.967823)
Supplement: Supplementary file 2 [file Table_2.docx]

Appendix S2. Weights matrix between the variables from the network analysis

| Variable | 1 | 2 | 3 | 4 | 5 | 6 | 7 | 8 | 9 | 10 | 11 | 12 | 13 |
| --- | --- | --- | --- | --- | --- | --- | --- | --- | --- | --- | --- | --- | --- |
| 1. Cognitive Disorganisation |  | .11 | .10 | .08 | .09 | .00 | .12 | -.04 | -.03 | .18 | .00 | .01 | .28 |
| 2. Depressive Experience |  |  | .08 | .19 | .04 | -.13 | .38 | .00 | -.01 | .11 | .12 | .15 | .00 |
| 3. Depressive Symptoms |  |  |  | .10 | .00 | .00 | .07 | .03 | .00 | .18 | .31 | .00 | .05 |
| 4. Impulsive Non-Conformity |  |  |  |  | .07 | .00 | .04 | -.02 | .03 | .12 | .02 | .05 | .10 |
| 5. Introvertive Anhedonia |  |  |  |  |  | -.08 | -.04 | -.18 | .05 | .07 | .06 | -.07 | -.01 |
| 6. Life Satisfaction |  |  |  |  |  |  | .05 | .33 | .00 | -.41 | .00 | .03 | .03 |
| 7. Manic Experience |  |  |  |  |  |  |  | -.05 | -.09 | -.04 | .04 | .29 | .04 |
| 8. Meaning in Life |  |  |  |  |  |  |  |  | .22 | .11 | -.02 | .10 | .04 |
| 9. Paranormal Belief |  |  |  |  |  |  |  |  |  | .05 | .10 | .16 | .27 |
| 10. Perceived Stress |  |  |  |  |  |  |  |  |  |  | .18 | -.05 | -.01 |
| 11. Somatic Complaints |  |  |  |  |  |  |  |  |  |  |  | .04 | .00 |
| 12. Transliminality |  |  |  |  |  |  |  |  |  |  |  |  | .42 |
| 13. Unusual Experiences |  |  |  |  |  |  |  |  |  |  |  |  |  |
