# Supplementary material for: Paranormal belief, cognitive-perceptual factors, and well-being: A network analysis
Source: Front Psychol. 2022 Sep 15;13:967823. doi: 10.3389/fpsyg.2022.967823 (PMC9521162; doi:10.3389/fpsyg.2022.967823)
Supplement: Supplementary file 3 [file Data_Sheet_1.PDF]

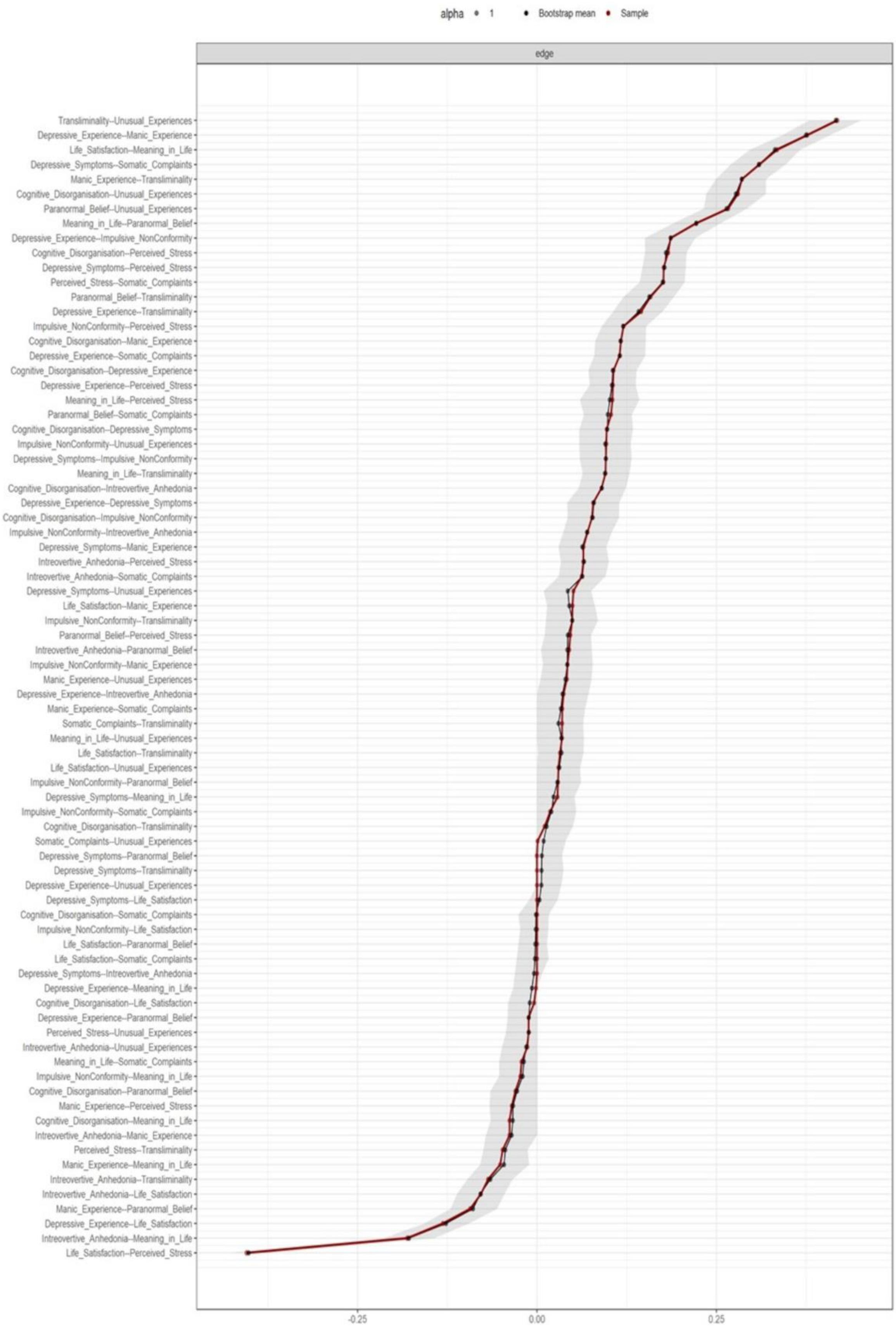

Appendix S3. Bootstrapped confidence intervals (CIs) of the edge weights in the network.
Note. The red line indicates the edge weight values, and the gray area indicates the 95% CIs.
