# Supplementary figures and images for: Paranormal belief, cognitive-perceptual factors, and well-being: A network analysis
Source: Front Psychol. 2022 Sep 15;13:967823. doi: 10.3389/fpsyg.2022.967823 (PMC9521162; doi:10.3389/fpsyg.2022.967823)

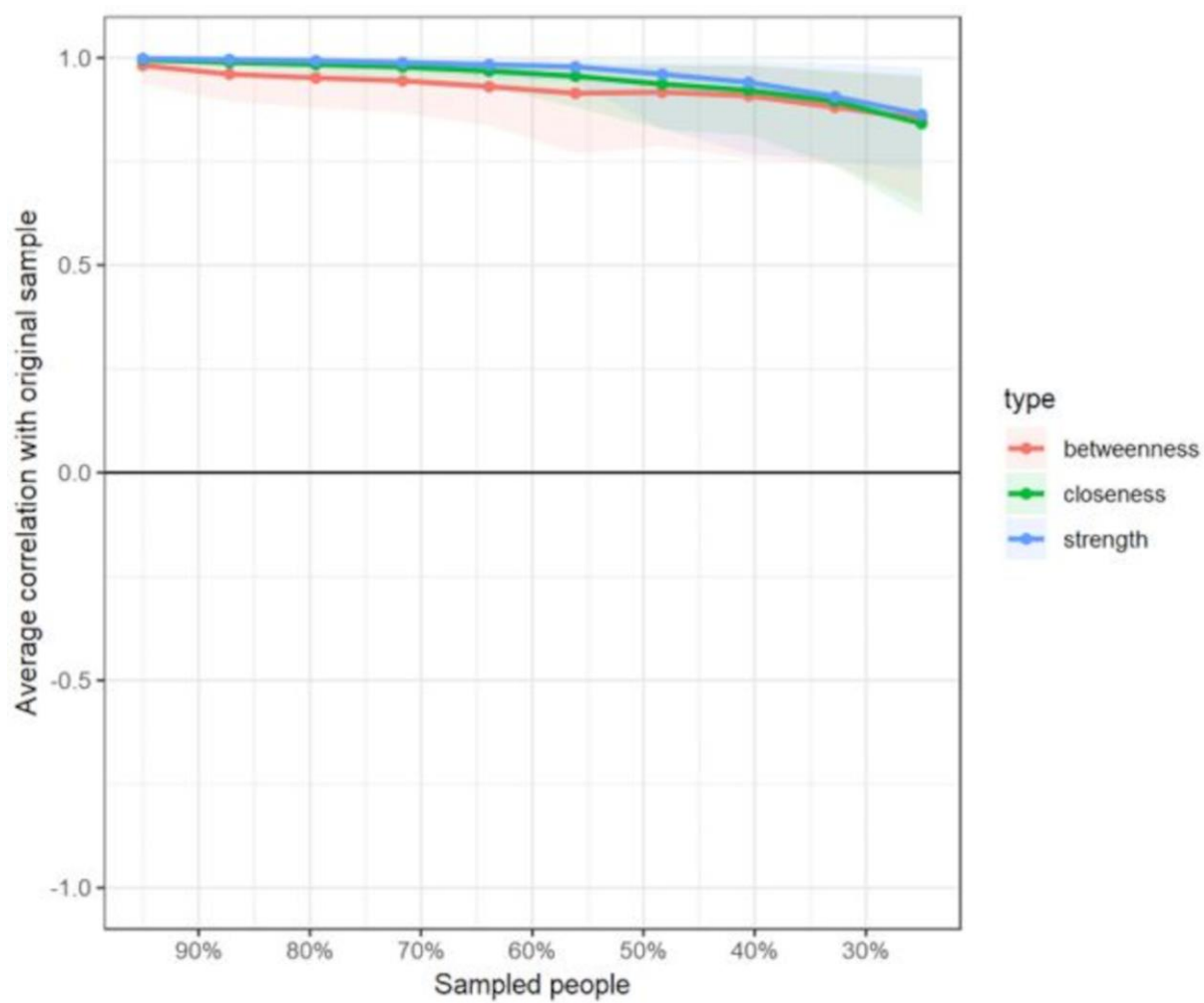

Appendix S4. Stability of central indices

Supplement: Supplementary file 4 [file Data_Sheet_2.PDF]
